# Beri-Beri

**Published:** 1909-04-24

**Authors:** 


### Tropical Diseases.

### BERI-BERI.

Of the many different diseases specially found in tropical climates none perhaps is so obscure ætiologically as beri-beri. The latter may be defined as an endemic peripheral neuritis often associated with an extreme grade of dropsy and generally with implication of the heart. It is responsible for a very large annual mortality and morbidity in the Straits Settlements and other parts of the East. Many theories have from time to time been advanced as to its cause; for example, that it is due to eating diseased rice, that it is due to a staphylococcus, that it is a local disease due to emanations from the soil, that it is a primary disease of the duodenum, toxins elaborated here being absorbed into the system and so causing the degenerative changes in the peripheral and other nerves.

Lately the first of these ideas—the rice theory—has been advanced again by Dr. Braddon, State Surgeon of Negri Sembilan in the Federated Malay States, and he tries to show that it is only certain kinds of rice that are responsible for the production of the disease. Similar instances with other cereals are not unknown. For example, pellagra is due to eating diseased maize; ergotism to eating diseased rye; and "lathyrism" is a disease brought on by eating various kinds of *lathyrus* (chick pea or pulse). Braddon describes the chief ways of preparing rice in the East thus:—

I. Fresh Rice: the method of the Peninsula Malays. The grain, dried in the sun, is garnered in dark bins, and from this store sufficient for the day's use is taken, and hulled by pounding it with a wooden pestle in a wooden mortar. Before cooking, the grain is washed carefully in water: this removes dust and other particles. Grain so prepared is said to be innocuous: the Malays who eat it do not suffer from beri-beri.

II. Uncured, stale White Rice: the grain collected from the natives is taken to mills and here is thoroughly dried, sifted to remove all foreign bodies, husked, hulled, or shelled, winnowed and screened. The completely stripped grain is next scoured, polished, and sized. The rice now, white, smooth, and clean, is distributed to different areas, and is ready for food. This kind of rice is dangerous to eat, and will in time produce beri-beri.

III. Cured Rice (the Indian method): Major Buchanan (quoted by Braddon) gives the details of this as follows: The rice is first soaked in water for about 12 hours; it is then put in earthen or iron vessels, mixed with water, and heated on a slow fire till the grains are burst. The boiled rice is next dried in the sun, and when dry the husks easily separate in the winnowing. In this method heat is used, and the grain is sterilised while in the husk, and so protected from the possible spread of any specific organism originally present on or in any of the seeds. Rice prepared in such a manner Braddon states is harmless. He brings forward a carefully selected series of statistics to support his contentions, and recent experiments with the two kinds of rice as food confirm his views.

